# Annotations

**Published:** 1903-03-14

**Authors:** 


### Annotations.

### The London Hospital.

There is one special ground upon which the London Hospital is entitled to claim the generous support of residents in wealthier districts than Whitechapel. We have evidence to show that of 13,160 in-patients admitted to the wards during 1902, in round numbers 6,000, or nearly one-half, resided within a radius of one mile from the hospital gates. Of the remainder 2,000, or about 30 per cent. resided within a two-mile radius, and 1,250, or just under one-fourth, within, and 4,000, *i.e.*, the remaining three-fourths, without the four-mile radius. These returns exhibit the work in its most favourable aspects, for the figures emphasise the fact, that the London Hospital provides especially for the poor population in its immediate vicinity. Great cities cannot do without the humbler workers, and they ought to receive marked consideration at the hands of the wealthier residents, who without them would find city life frequently unpleasant and sometimes impossible. On the ground, therefore, that the London Hospital is the hospital of the poor in East London, we would urgently commend it to the support of all classes who have the means to contribute something each year to one of the great hospitals. The Lord Mayor's appeal has been issued, and we hope it will receive liberal support. We are most anxious that the London Hospital should obtain all the money it requires without delay, and we know nothing better calculated to make the Lord Mayor's appeal popular, than a knowledge of the facts we have just stated in support of the London Hospital. It is not easy for the man in the street to determine the relative merits of the claimants for his bounty from the letters of appeal which find their way to his breakfast table each morning. He is often too much occupied to make a personal investigation, but in the case of the London Hospital no investigation is necessary so far as the urgency of the needs is concerned. Its popularity and usefulness to the poorer residents in Whitechapel and the East End of London afford the most practical guarantees, that everybody is justified in sending a contribution to the quinquennial fund. At least £200,000 should be forthcoming, and less than half this amount has yet been subscribed. In the present season of the churches' year, a further £100,000 at least, should be speedily given in support of London's largest hospital. We shall hope to hear that all the money needed has been subscribed by Easter at the latest.

### The Ill-Health of Darwin.

Any influence which tends to limit or mar the working powers of the world's master minds cannot but be of interest and concern to all thinking people. It is not surprising, therefore, to find that two writers have recently attempted to solve the perplexities which surrounded the disorder from which Darwin suffered, and which, as is shown in his "Life and Letters," proved so serious a hindrance to the conduct of his scientific work. Dr. George M. Gould, in his very ingeniously constructed *Biographic Clinics*, by means chiefly of quotations and extracts, tells the clinical history of the origin and course of Darwin's ailment, and seeks to show that his symptoms, vomiting and other functional digestional disorders, insomnia, vertigo, headache, apathy, and neurasthenia, were dependent on an ocular refractive anomaly, now known as compound hyperopic astigmatism. Another recent essayist, Dr. W. W. Johnston, considers that the discomforts, fatigues, and mental overstrain of the *Beagle* voyage can fairly be set down as the initial cause of Darwin's subsequent invalidism. He considers the case to have been one of chronic neurasthenia of a severe grade, due first to the overstrain of Darwin's early voyage, and second to his life of hard intellectual work. But where doctors differ who shall decide? In studying the lives of distinguished students and learned workers one cannot but be struck with the lack of scientific precision and logical insight with which they often view their own ailments and not infrequently themselves undertake their alleviation. Indeed, it may safely be said that, at least in many cases, a poor patient attending an out-patient department of a London hospital is more likely to obtain an early and accurate diagnosis and be set on the road leading to permanent relief than a hesitating and self-willed *savant*. The latter oftentimes more readily follows the suggestions of a fellow-worker or obeys the mandates of a quack rather than accept the services of a skilled physician.

### The Parasitic Origin of Cancer.

Much activity still continues in the unravelling of the mystery which surrounds the etiology of malignant disease. Mr. Roger Williams, in a reprint from "Supplement of Twentieth-century Practice of Medicine," vol. xxi., 1903, has recently supplied a useful summary of the work relating to the parasitic origin of cancer. He shows that malignant tumours are singularly prone to bacterial infection, owing to their feeble vitality, which is often a consequence of defective blood supply. In their young and nascent condition non-ulcerated malignant tumours seem always to be quite free from bacteria. But although many organisms may find a suitable *habitat* in malignant tumours, none of them fulfil the requirements necessary for the establishment of their etiological as true "cancer microbes." And with regard to the influence of protozoa, the evidence in favour of a causal connection with cancer is almost entirely histological, for the results of culture and inoculation experiments have established nothing definite. Certain blastomycetes, it is now generally admitted, are pathogenic for man and animals and may even cause lesions which in their gross features resemble malignant formations, but when carefully examined these mycotic pseudoplasms are usually found to differ markedly from true malignant growths, both structurally and in their clinical features. Many pathologists have succeeded in obtaining pure cultures of blastomycetes from human malignant growths; but considering the richness of these tumours in sugar-forming materials, it would indeed be strange if they were not more prone to this kind of infection than the normal tissues of the body. Inoculation with these organisms certainly fails to produce malignant disease, and no satisfactory evidence is at present forthcoming to favour the idea that cancer is a contagious disease.

